# Colorectal Cancer Linkage on Chromosomes 4q21, 8q13, 12q24, and 15q22

**DOI:** 10.1371/journal.pone.0038175

**Published:** 2012-05-31

**Authors:** Mine S. Cicek, Julie M. Cunningham, Brooke L. Fridley, Daniel J. Serie, William R. Bamlet, Brenda Diergaarde, Robert W. Haile, Loic Le Marchand, Theodore G. Krontiris, H. Banfield Younghusband, Steven Gallinger, Polly A. Newcomb, John L. Hopper, Mark A. Jenkins, Graham Casey, Fredrick Schumacher, Zhu Chen, Melissa S. DeRycke, Allyson S. Templeton, Ingrid Winship, Roger C. Green, Jane S. Green, Finlay A. Macrae, Susan Parry, Graeme P. Young, Joanne P. Young, Daniel Buchanan, Duncan C. Thomas, D. Timothy Bishop, Noralane M. Lindor, Stephen N. Thibodeau, John D. Potter, Ellen L. Goode, for the Colon CFR

**Affiliations:** 1 Departments of Health Sciences Research, Laboratory Medicine and Pathology, and Medical Genetics, Mayo Clinic College of Medicine, Rochester, Minnesota, United States of America; 2 Department of Epidemiology, University of Pittsburgh, Pittsburgh, Pennsylvania, United States of America; 3 Department of Preventive Medicine, University of Southern California, Los Angeles, California, United States of America; 4 University of Hawaii Cancer Center, Honolulu, Hawaii, United States of America; 5 Department of Molecular Medicine, Beckman Research Institute of the City of Hope, Duarte, California, United States of America; 6 Faculty of Medicine, Memorial University of Newfoundland, St. Johns, Newfoundland, Canada; 7 Department of Surgery, University of Toronto, Toronto, Ontario, Canada; 8 Cancer Prevention Program, Fred Hutchinson Cancer Research Center, Seattle, Washington, United States of America; 9 Departments of Public Health and Medicine, University of Melbourne, Victoria, Australia; 10 Colorectal Medicine and Genetics and Department of Medicine, University of Melbourne, The Royal Melbourne Hospital, Victoria, Australia; 11 New Zealand Familial GI Cancer Registry, Auckland City Hospital, Auckland, New Zealand; 12 Flinders Centre for Cancer Prevention and Control, Flinders University, Adelaide, Australia; 13 Familial Cancer Laboratory, Queensland Institute of Medical Research, Queensland, Australia; 14 University of Leeds, Leeds Institute of Molecular Medicine, Leeds, United Kingdom; 15 Centre for Public Health Research, Massey University, Wellington, New Zealand; 16 Department of Gastroenterology, Middlemore Hospital, Auckland, New Zealand; The Chinese University of Hong Kong, Hong Kong

## Abstract

A substantial proportion of familial colorectal cancer (CRC) is not a consequence of known susceptibility loci, such as mismatch repair (MMR) genes, supporting the existence of additional loci. To identify novel CRC loci, we conducted a genome-wide linkage scan in 356 white families with no evidence of defective MMR (i.e., no loss of tumor expression of MMR proteins, no microsatellite instability (MSI)-high tumors, or no evidence of linkage to MMR genes). Families were ascertained via the Colon Cancer Family Registry multi-site NCI-supported consortium (Colon CFR), the City of Hope Comprehensive Cancer Center, and Memorial University of Newfoundland. A total of 1,612 individuals (average 5.0 per family including 2.2 affected) were genotyped using genome-wide single nucleotide polymorphism linkage arrays; parametric and non-parametric linkage analysis used MERLIN in *a priori*-defined family groups. Five lod scores greater than 3.0 were observed assuming heterogeneity. The greatest were among families with mean age of diagnosis less than 50 years at 4q21.1 (dominant HLOD = 4.51, α = 0.84, 145.40 cM, rs10518142) and among all families at 12q24.32 (dominant HLOD = 3.60, α = 0.48, 285.15 cM, rs952093). Among families with four or more affected individuals and among clinic-based families, a common peak was observed at 15q22.31 (101.40 cM, rs1477798; dominant HLOD = 3.07, α = 0.29; dominant HLOD = 3.03, α = 0.32, respectively). Analysis of families with only two affected individuals yielded a peak at 8q13.2 (recessive HLOD = 3.02, α = 0.51, 132.52 cM, rs1319036). These previously unreported linkage peaks demonstrate the continued utility of family-based data in complex traits and suggest that new CRC risk alleles remain to be elucidated.

## Introduction

Colorectal cancer (CRC) is the third most common cancer and the third leading cause of cancer death in the United States. Approximately 141,210 new cases and 49,380 deaths from CRC were expected in the United States in 2011 [1]. Family history is a consistent risk factor [2]; without CRC family history, the lifetime risk for an individual above the age of 50 years is 5% to 6%, yet this can be as high as 20% when there are first- or second-degree relatives with CRC [3]–[5], and reaches 80% to 100% in familial syndromes [6]. Lynch syndrome represents up to 5% of CRCs and results from germline mutations in one of several DNA mismatch repair (MMR) genes (*MLH1*, *MSH2*, *MSH6* and *PMS2* [7]. MMR mutations result in a defective mismatch repair (dMMR) tumor phenotype manifested by absence of MMR protein expression [8], [9] and DNA microsatellite instability (MSI-H). Segregation analyses excluding Lynch syndrome families suggest that additional loci for CRC susceptibility exist [5].

To identify novel loci, case-control association studies and family-based linkage analyses serve as complementary approaches. At least fifteen, common low-penetrance risk alleles have emerged from genome-wide association studies (GWAS) including at 1q41 (rs6691170, *DUSP10*) [10], 3q26.2 (rs10936599, *MYNN*) [10], 8q23.3 (rs16892766, *EIF3H*) [11], 8q24 (rs6983267) [12], [13], 9p24 (rs719725) [14], 10p14 (rs10795668) [11], 11q23 (rs3802842) [15], 12q13.13 (rs11169552) [10], 14q22.2 (rs4444235, *BMP4*) [16], 15q13 (rs4779584) [15], 16q22.1 (rs9929218, *CDH1*) [16], 18q21 (rs4939827, *SMAD7*) [17], 19q13.1 (rs10411210, *RHPN2*), 20p12.3 (rs961253) [16], and 20q13.33 (rs4925386, *LAMA5*) [10]. Studies of CRC linkage in multi-case families or affected sibling pairs have reported evidence of rare, high-risk variants in several genetic regions including 3q21-24, 7q31, 9q22-31, and 11q23 [18]–[26]. Most linkage studies to date have utilized fewer than 100 families, and only two studies excluded dMMR families [18], [26].

Here, we describe a genome-wide linkage scan of 356 white families without evidence of dMMR using family groups defined by age at diagnosis, ascertainment method, and number of affected family members. This represents the largest linkage study of proficient MMR (pMMR) CRC families to date and suggests novel regions with evidence of high-penetrance loci.

## Materials and Methods

### Ethics Statement

All participants gave written informed consent. Ontario Cancer Research Ethics Board, University of Southern California Institutional Review Board, University of Melbourne Institutional Review Board, University of Hawaii Institutional Review Board, Mayo Clinic Institutional Review Board, Fred Hutchinson Cancer Research Center Institutional Review Board, Memorial University of Newfoundland Institutional Review Board and City of Hope Institutional Review Board approved protocols.

### Ascertainment and Collection of Families

A total of 578 linkage-informative families were identified having at least two affected individuals diagnosed with invasive CRC in sibling, half-sibling, cousin, grand-parental, or avuncular pairs [27], absence of sequence-confirmed Lynch Syndrome and MYH-associated polyposis [28], and absence of medical-record-confirmed familial adenomatous polyposis.

The majority of families (N = 480) were from the Colon Cancer Family Registry multi-site NCI-supported consortium (Colon CFR) ascertained between 1997 and 2007 by Cancer Care Ontario (Toronto, Canada), a University of Southern California Consortium (Los Angeles, CA), a University of Melbourne Consortium (Victoria, Australia), the University of Hawaii (Honolulu, HI), the Mayo Clinic (Rochester, MN), and the Fred Hutchinson Cancer Research Center (Seattle, WA) [28]. All study sites ascertained population-based families, although varying sampling schemes based upon age and/or family history were used. Clinic-based families were ascertained by the University of Melbourne Consortium (through family cancer clinics in Adelaide, Perth, Sydney, Brisbane, and Melbourne, Australia and Auckland, New Zealand), the University of Southern California Consortium (through the Cleveland Clinic), and the Mayo Clinic. Epidemiologic data, blood samples, tumor blocks, and pathology reports were collected on all participants with CRC at each site, using standardized core protocols.

Clinic-based families from a City of Hope consortium (N = 59) were recruited between 1998 and 2005 at the City of Hope (Duarte, CA), Tufts University (Medford, MA), the University of Pittsburgh (Pittsburgh, PA), Northwestern University (Chicago, IL), the University of Wisconsin (Madison, WI), Vanderbilt University (Nashville, TN), the University of South Florida/Moffitt Cancer Center (Tampa, FL), Maine Medical Center (Portland, ME), and Rose Medical Center (Denver, CO). White CRC cases older than 18 years of age, who had at least one living sibling diagnosed with CRC, were enrolled. Blood samples, pathology reports, and a brief questionnaire focused on ethnicity and family history were collected on all cases.

Population-based and clinic-based families (N = 39) from Newfoundland and Labrador, Canada were obtained at Memorial University of Newfoundland as previously described [29], [30]. Briefly, pathologically confirmed cases diagnosed under the age of 75 years were enrolled via the provincial tumor registry between 1997 and 2003. Epidemiologic data including family history and risk factors, blood samples, tumor tissue, and pathology reports were collected. Clinic-based families were contacted following referrals to the high-risk cancer clinic of the provincial Medical Genetics Program.

### SNP Genotyping and Quality Control

We genotyped all available affected individuals within each family, as well as key unaffected individuals, including siblings, children, and spouses of deceased affected individuals; parents of affected siblings; grandparents of affected cousins; and other individuals useful for estimation of phase [31]. Single nucleotide polymorphism (SNP) genotyping was conducted using the Affymetrix 10K 2.0 array (Affymetrix, Santa Clara, CA) for 327 families (1,753 individuals) and the Illumina Infinium Linkage 12 bead array (Illumina, San Diego, CA) for 251 families (1,001 individuals) following manufacturers' protocols [32], [33]. A CEU trio (Coriell Institute for Medical Research, Camden, NJ, USA) was included in each 96-well plate.

Hardy-Weinberg equilibrium (HWE) testing relied on mean p-values from exact testing of 100 random samples of one individual per pedigree. SNPs were excluded with unknown genetic position (n = 147) (build 36.3), call rate <95% (n = 1,076), minor allele frequency (MAF) <1% (n = 377), HWE p-value<0.001 (n = 17), duplicate concordance <95% (n = 10), or Mendelian error in >2% of families (n = 4). We also excluded SNPs to reduce LD (r^2^>0.10) (n = 4,512) in order to minimize false-positive linkage findings (Table S1) [34]. For 10,091 unique SNPs remaining combined across arrays, genetic maps were created using the Rutgers linkage-physical map v.2 [35] and converted from Kosambi to Haldane distance.

### Family Exclusions

We aimed to analyze white families without relationship errors and without evidence of MMR deficiency. Self-reported family structures were confirmed via evaluation of Mendelian inheritance using PREST [36] and Pedcheck [37] based on SNP data. Where probable sample switches or non-paternities were found, family structures were altered (nine sibships changed to half-sibships) or excluded (34 families excluded). We used EIGENSTRAT [38] to estimate ethnicity for individuals with missing self-reported ethnicity, verify ethnic similarity among related individuals, and exclude families with individuals clustering outside the large self-reported white cluster (43 families were excluded, Figure S1).

MMR proficiency was evaluated using MSI testing, immunohistochemical (IHC) analysis, and LOD scores at known MMR loci. MSI testing of Colon CFR and Newfoundland families was performed on multiple family members using paired normal and tumor DNA isolated from formalin-fixed, paraffin-embedded (FFPE) material [39]. Ten markers were tested (mono-nucleotide markers BAT25, BAT26, BAT34C4, and BAT40; di-nucleotide markers ACTC, D5S346, D10S197, D17S250, and D18S55; and complex repeat MYCL), and four unequivocal results were required. Eighty-nine families with at least one MSI-H tumor were excluded. IHC analysis of Colon CFR and Newfoundland families for MLH1, MSH2, MSH6, and PMS2 expression was performed on FFPE samples, as previously described [39]. IHC staining across all sites was done at three centers, and pathologist interpretation was conducted blind to MSI status. Forty-one families in which at least one tumor showed protein loss were excluded. Finally, we excluded an additional 15 families with dominant LOD scores >0.4 within 20 kb surrounding *MSH2*, *MLH1*, *MSH6*, *PMS2*, *PMS1*, *MSH3*, or *MLH3* (linkage methods described below). Thus, 356 white families with no evidence of MMR deficiency were included in the analysis.

### Linkage Analysis

Multipoint parametric and nonparametric linkage analyses used MERLIN version 1.1.2 [40]; dominant and recessive models were based on a prior segregation analysis (Table S2) [5]. Parametric linkage in the presence of heterogeneity was assessed using heterogeneity LOD (HLOD) scores, and the proportion of families linked to each locus (α) was estimated using HOMOG [41]. Non-parametric Kong & Cox LOD (NPL) scores from the linear model were computed along with S_all_ statistics [42], [43]. As has been useful for other cancers [44], [45], we sought to improve power by increasing genetic homogeneity using family sub-groups defined *a priori* based on presumed genetically relevant characteristics. Thus, family groups were based on mean age at diagnosis (<50 years, ≥50 years), ascertainment scheme (population-based, clinic-based, or unknown), and number of affected individuals (2, 3, 4 or more). Likelihood ratio testing evaluated heterogeneity of linkage across the independent subsets of each subgroup factor (i.e., age at diagnosis, ascertainment scheme, and number of affected individuals).

### Association Analysis

In key regions identified by linkage analysis, we also performed association testing among an additional 1,136 cases (343 family history positive and 793 family history negative cases with and without 1^st^ degree relative with CRC, respectively) and 997 controls from population-based collections of the Colon CFR who were genotyped using the Illumina 1M/1M Duo SNP array, as described previously [46]. Logistic regression estimated association between genotype and CRC risk adjusted for age, gender, study site, and four principal components representing ancestry [46]. A quantile-quantile (Q-Q) plot of genome-wide observed versus expected test statistics indicated no evidence of inflation (λ = 0.938) [46].

## Results

This collection of white CRC families with no evidence of dMMR consisted of 277 families from the Colon CFR, 48 families from the City of Hope consortium, and 31 families from Newfoundland. A total of 1,612 individuals were successfully genotyped including, on average, five individuals per family (range, 2–10, mean 2.2 affected and 2.8 unaffected individuals). The mean age at diagnosis was 59.7 years (range, 36–79) and 56.2 years (range, 31–74) among population- and clinic-based families, respectively. The majority of families had two affected members (56%) and an older (>50 years) mean age at diagnosis (84%). MSI data were available on 224 families (209 MSS and 15 MSI-L), and IHC data were available on 255 families and showed no evidence of MMR deficiency (Table 1). Both MSI and IHC data were available on 190 families. Sixty-seven families were not tested but had a LOD<0.04 within 20 kb surrounding *MSH2*, *MLH1*, *MSH6*, *PMS2*, *PMS1*, *MSH3*, or *MLH3*.

**Table 1:** Characteristics of 356 White Colorectal Cancer Families with No Evidence of Defective Mismatch Repair, N (%).

		Ascertainment Method		
		Population-basedN = 189	Clinic-basedN = 88	Unknown^a^N = 79
Mean Age of Diagnosis	<50 years	24 (13%)	19 (22%)	15 (19%)
	≥50 years	165 (87%)	69 (78%)	64 (81%)
Number of Affected Individuals	2	115 (61%)	26 (30%)	59 (75%)
	3	51 (27%)	27 (31%)	11 (14%)
	4–10	23 (12%)	35 (40%)	9 (11%)
MSI^b^	MSS	119 (63%)	61 (69%)	29 (37%)
	MSI-L	9 (5%)	6 (7%)	0
	Unknown^d^	61 (32%)	21 (24%)	50 (63%)
IHC Expression of MMR Genes^c^	No Loss	147 (78%)	77 (88%)	31 (39%)
	Unknown^d^	42 (22%)	11 (13%)	48 (61%)

a Ascertainment method not reported.

b Microsatellite stability of the tumor; MSS indicates that the tumor was microsatellite stable; MSI-L indicates that the tumor had low microsatellite instability; Unknown indicates that the tumor stability status was not available.

c Mismatch repair status of the tumor by immunohistochemical analysis; No loss indicates that the tumor showed complete presence of protein expression of all the MMR genes tested (MLH1, MSH2, MSH6, and PMS2); Unknown indicates that the tumor MMR-expression status was not available.

d Unknown for both MSI and IHC on 67 families is due to not being tested but had a LOD<0.04 within 20 kb surrounding *MSH2*, *MLH1*, *MSH6*, *PMS2*, *PMS1*, *MSH3*, or *MLH3*.

**Figure 1:**
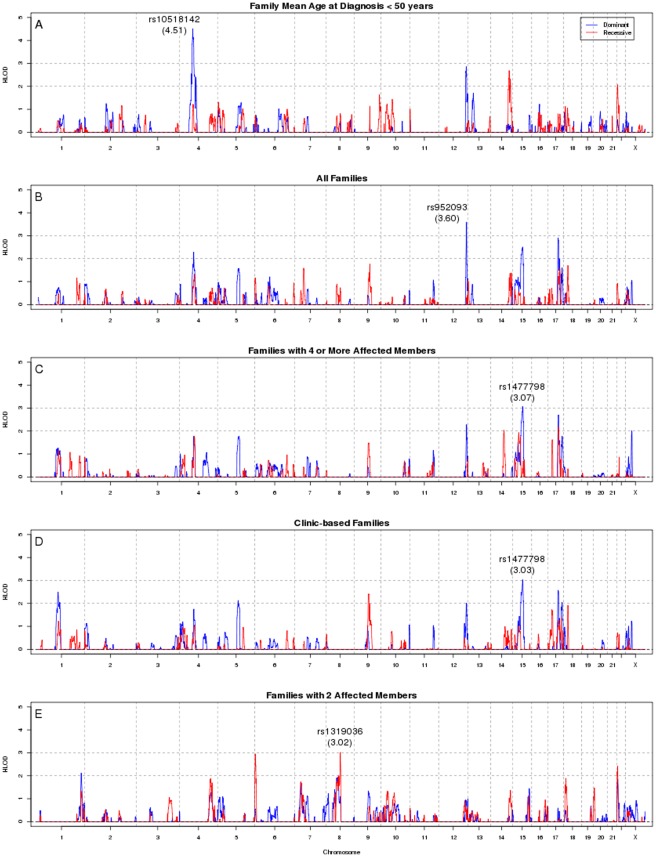
Genome-wide Linkage Scans of White pMMR Family Groups with HLOD Score>3.0. HLOD scores from genome-wide linkage scan of five white pMMR family subgroups. The blue line represents HLODs under the dominant model and the red line represents the HLODs under the recessive model. Maximum observed HLODs>3.0 (in parenthesis) are labeled with the nearest SNP in four regions. (A) Family mean age at diagnosis <50 years (N = 58). (B) All families (N = 356). (C) Families with four or more affected members (N = 67). (D) Clinic-based families (N = 88). (E) Families with two affected members (N = 200).

Genome-wide linkage scans of nine family groups were conducted including analysis of all families and of subsets of families defined by age, ascertainment scheme, and number of affected individuals. Four regions in five family groups were observed with HLOD scores greater than 3.0 (Figure 1). The strongest result was based on analysis of 58 families with a mean age at diagnosis <50 years. In this group, we observed a dominant HLOD of 4.51 on chromosome 4q21.1 (145.40 cM, NPL = 2.52) with an estimated 84% of families linked (Table 2). The peak occurred at rs10518142 which is in intron 5 of *NAAA* encoding N-acylethanolamine acid amidase. The linkage region, defined as a 1-HLOD support interval, spanned 16.0 cM (8.7 Mb). This peak was not seen in older mean age at diagnosis families (Figure S2), although significant heterogeneity by mean age at diagnosis was not observed (LRT p = 0.35). Other regions of interest in families defined by age at diagnosis (HLOD>2.0) are provided in Table 3.

**Table 2:** Summary of Colorectal Cancer Linkage Results with HLOD Scores>3.0.

Family Group	N Families	Region	cM (Nearest SNP^a^, Mbp)	Nearest Gene (SNP location)	Model	HLOD (α)	NPL^c^
Mean Age at Diagnosis <50	58	4q21.1	145.40 (rs10518142, 77.1)	*NAAA* (intron)	Dominant	4.51 (0.84)	2.52
All Families	356	12q24.32	285.15 (rs952093, 127.4)	*TMEM132C* (intron)	Dominant	3.60 (0.48)	2.88
4+ Affected	67	15q22.31	101.40 (rs1477798, 64.2)	*MEGF11* (intron)	Dominant	3.07 (0.29)	1.03
Clinic-based	88	15q22.31	101.40 (rs1477798, 64.2)	*MEGF11* (intron)	Dominant	3.03 (0.32)	0.98
2 Affected	200	8q13.2	132.52 (rs1319036, 70.3)	*LOC100129096* b (intron)	Recessive	3.02 (0.51)	0.08

a Genotyped SNP nearest to the peak of the linkage region; distances are reported in bp based on the NCBI build 36.2 and Haldane cM.

b Pseudo-gene.

c Non-parametric Kong & Cox LOD.

**Table 3:** Summary of Colorectal Cancer Linkage Results with Maximum Observed HLOD Scores between 2.0 and 3.0.

Family Group	Linkage Region	cM (Nearest SNP^a^, Mbp)	Nearest Gene	SNP Location^b^ (distance, bp)	Model	HLOD (α)
All Families (N = 356)	4q21.21	154.41 (rs725826, 82.4)	*RASGEF1B*	3′ downstream (70,563)	Dominant	2.29 (0.29)
	15q22.31	101.40 (rs1477798, 64.2)	*MEGF11*	intron	Dominant	2.51 (0.20)
	17q23.2	143.49 (rs888115, 53.0)	*MSI2*	intron	Dominant	2.91 (0.37)
Mean Age at Diagnosis <50 (N = 58)	12q24.32	277.56 (rs2013160, 126.3)	*LOC644489* d	3′ downstream (40,332)	Dominant	2.86 (0.86)
	14q32.1	161.58 (rs1956716, 94.0)	*SERPINA12*	5′ upstream (18,174)	Recessive	2.68 (0.41)
	22q11.21	7.92 (rs4269007, 16.7)	*MICAL3*	intron	Recessive	2.07 (0.24)
Mean Age at Diagnosis ≥50 (N = 298)	15q22.33	106.55 (rs1822829, 65.3)	*AAGAB*	intron	Dominant	2.66 (0.27)
	17q23.2	143.50 (rs888115, 53.0)	*MSI2*	intron	Dominant	2.87 (0.42)
Population-based (N = 189)	7p14.1	89.98 (rs1949880, 39.0)	*POU6F2*	intron	Recessive	2.39 (0.38)
	13q12.11	3.88 (rs264729, 21.1)	*LOC100128060* d	3′ downstream (4,619)	Recessive	2.35 (0.40)
Clinic-based (N = 88)	1p21.3	217.34 (rs1144305, 96.3)	*LOC100132258* d	5′ upstream (207,801)	Dominant	2.50 (0.51)
	5q21.3	199.19 (rs1561350, 106.1)	*LOC345571* d	5′ upstream (231,963)	Dominant	2.13 (0.23)
	9q21.32	136.57 (rs918223, 85.6)	*GKAP1*	intron	Recessive	2.41 (0.32)
	12q24.32	285.15 (rs952093, 127.4)	*LOC100132385* d	3′ downstream (77,728)	Dominant	2.01 (0.38)
	17q23.2	143.49 (rs888115, 53.0)	*MSI2*	intron	Dominant	2.58 (0.41)
Unknown Ascertainment^c^ (N = 79)	13q13.3	46.30 (rs1170994, 35.5)	*DCLK1*	intron	Dominant	2.16 (1.00)
2 Affected (N = 200)	1q42.3	467.95 (rs1405633, 234.5)	*LOC343508*	5′ upstream (13,790)	Dominant	2.12 (1.00)
	6p25.3	2.63 (rs1055368, 852.0)	*EXOC2*	5′ upstream (214,441)	Recessive	2.95 (0.52)
	8q12.2	121.32 (rs1367972, 62.2)	*NPM1P6*	5 upstream (111,467)	Recessive	2.00 (0.51)
	22q11.21	8.62 (rs387399, 16.8)	*MIR648*	5′ upstream (2,433)	Recessive	2.42 (0.46)
3 Affected (N = 89)	5p14.1	77.05 (rs1966983, 31.8)	*PDZD2*	5′ upstream (53,152)	Dominant	2.02 (0.73)
	13q12-q13	8.88 (rs727081, 22.6)	*SGCG*	5′ upstream (22,892)	Recessive	2.07 (0.49)
4+ Affected (N = 67)	12q24.32	285.16 (rs952093, 127.4)	*TMEM132C*	intron	Dominant	2.28 (0.40)
	14q24.3	110.11 (rs1125221, 74.0)	*TMEM90A*	5′ upstream (20,025)	Recessive	2.03 (0.39)
	17q23.1	147.11 (rs1296279, 55.3)	*TUBD1*	intron	Dominant	2.70 (0.36)
	Xp11.3	119.99 (rs1375329, 43.8)	*LOC643167*	3′ downstream (23,566)	Dominant	2.01 (0.41)

a Genotyped SNP nearest to the peak of the linkage region; distances are reported based on the NCBI build 36.2 and Haldane cM.

b SNP location and the distance in bp is given in regards to the nearest gene.

c Ascertainment method not reported.

d Pseudo-gene.

The second strongest linkage peak occurred in analysis of all families (N = 356) at 12q24.32 with a maximum dominant HLOD of 3.60 (285.15 cM, NPL = 2.88) and an estimated 48% of families linked (Table 2). The peak SNP, rs952093, resides in intron 1 of *TMEM132C* encoding transmembrane protein 132C; the equivalent of a 1-HLOD interval defined a 14 cM (1.3 Mb) region. Three suggestive regions in analysis of all families (HLOD>2.0) were seen on chromosomes 4, 15, and 17 (Figure 1; Table 3), including a region near to the 4q21.1 peak seen in younger age at diagnosis families.

Additional linkage peaks with HLODs just over 3.0 were observed on chromosome 15q22.31 (101.40 cM, rs1477798) among 67 families with four or more affected individuals and among 88 clinic-based families (Figure 1). Among families with at least four affected members, a dominant HLOD of 3.07 was observed (α = 0.29, NPL = 1.03), and among clinic-based families a dominant HLOD 3.03 was seen (α = 0.32, NPL = 1.03). Thirty-five families contributed to both analyses (i.e., clinic-based families with four or more affected individuals) (Table 4); analysis of these revealed a dominant HLOD of 3.15 (α = 0.35, NPL = 1.88). Of note, this region was also suggested by analysis of all families (HLOD = 2.51, α = 0.20, Table 3). This peak was not seen in analysis of smaller families, population-based families, or families with unknown ascertainment, although significant heterogeneity by family size or ascertainment scheme was not observed (all LRT p's>0.10). rs1477798 is in intron of *MEGF11* which encodes multiple EGF-like-domains 11.

**Table 4:** The overlap in the distribution of family groups.

Family Group	Mean Age at Diagnosis <50	Mean Age at Diagnosis ≥50	Population-based	Clinic-based	2 Affected	3 Affected	4+ Affected
Mean Age at Diagnosis <50	***63***	0	34	20	9	28	20
Mean Age at Diagnosis ≥50	0	***293***	166	69	58	161	68
Population-based	34	166	***200***	0	0	115	26
Clinic-based	20	69	0	***89***	0	51	27
2 Affected	9	58	0	0	***67***	23	35
3 Affected	28	161	115	51	23	***189***	0
4+ Affected	20	68	26	27	35	0	***88***

An additional HLOD over 3.0 was observed in recessive analysis of 200 families with only two affected family members (Figure 1, Table 2). On 8q13.2, a recessive HLOD of 3.02 was seen at rs1319036 (intron in pseudo-gene *LOC100129096*, α = 0.51, NPL = 0.08). Linkage assuming a recessive mode of inheritance is consistent with an affected sibling pair family structure. This region was not highlighted in analysis of larger families (Table 3), although significant heterogeneity by family size was not observed (LRT p = 0.94). Another region of note is 17q23.2 which revealed a dominant HLOD of 2.91 among all families (143.49 cM, α = 0.37) and dominant HLOD of 2.87 (143.50 cM, α = 0.42) among 298 families with mean age of diagnosis ≥50 years (Table 3); the peak SNP rs888115 is in intron 4 of *MSI2* which encodes musashi homolog 2 (Drosophila). Additional linkage results are provided in Figure S2. A second recessive model linkage peak downstream of 8q13.2 was observed in the same families with two affected members (HLOD = 2.0, α = 0.51) on 8q12.2. These two nearby peaks were 11.2 cM (8.1 Mb) apart.

Finally, we analyzed association within the 1-HLOD-support intervals surrounding each linkage peak with HLOD>3.0 using additional Colon CFR cases (N = 1,176) and controls (N = 997). In 4q21.21, which showed evidence of linkage in younger age at diagnosis families, the linkage SNP rs10518142 showed no evidence of association; however, rs12643573, which is 2 cM downstream, showed some evidence of association (OR 1.64, p = 5.4×10^−5^; family history positive OR 1.82, p = 1.0×10^−4^) (Figure S3). At rs1477798 in 15q22.31 which showed evidence of linkage in clinic-based, larger families, a nominally significant case-control association was observed (OR 1.16, p = 0.04) which was modestly strengthened for cases with CRC family history (OR 1.24, p = 0.03); however, no significant difference in risk by family history was observed and associations were far from genome-wide significant. No other associations of note were observed.

## Discussion

Results of this genome-wide linkage scan provide strong evidence for four previously- unreported CRC susceptibility loci. Notably, we identified a region at 4q21.1 among families with younger mean age at diagnosis (dominant HLOD = 4.51) and estimated that 84% of these families were linked. The 1-HLOD-support interval of this region, 16 cM (139 cM–155 cM) spanning 8.7 Mb, contains multiple known genes including *NAAA*. *NAAA* encodes an N-acylethanolamine-hydrolyzing enzyme and is shown to be expressed in variety of human tissues including colon [47]. Many of the genes upstream and downstream of *NAAA* are members of the chemokine family that are clustered in 4q12-21 region. The CXC chemokines modulate tumor behavior by regulation of angiogenesis, activation of a tumor-specific immune response, and direct stimulation of tumor proliferation in an autocrine or paracrine fashion [48].

Among all families, evidence for linkage was seen at 12q24.32 (HLOD = 3.60) with an estimated 48% of families linked to this locus (1-HLOD-support interval of 14 cM [276 cM–290 cM] spanning 1.3 Mb). This region contains four known genes (*TMEM132C*, *SLC15A4*, *GLT1D1*, and *TMEM132D*), four hypothetical genes (*LOC100128554*, *LOC387895*, *LOC440117*, and *FLJ37505*), and one microRNA (*MIR3612*). The four known genes in this region are conserved in dog, mouse, and chicken and, in some cases, zebrafish and Arabidopsis. One of these transmembrane proteins (TMEM132D) is known to be expressed in mature oligodendrocytes [49], but little else is known about either function or pathology, as is also true of *GLT1D1* (glycosyltransferase 1 domain containing 1) in humans. Members of the SLC15 (solute carrier family 15) family are electrogenic transporters of short-chain peptides into a variety of cells [50]. Evidence for linkage at 15q22.31, with a 1-HLOD-support interval of 38 cM (78 cM–116 cM) spanning 12.9 Mb, was particularly evident among families enrolled at high-risk clinics or with four or more affected individuals (dominant HLOD = 3.15). This is a large gene-rich region and contains many known genes including *MEGF11* and *RAB11A*. Very little is known about *MEGF11* [51]. *RAB11A* is a RAS oncogene family member expressed in tumor cell lines and suggested to be involved in membrane trafficking [52]. Finally, among families with only two affected individuals, the 1-HLOD-support interval of 12 cM (126 cM–138 cM) spans 5 Mb (8q13.2, recessive HLOD = 3.02) and contains mostly pseudogenes. Notably, *SULF1* in this region has been suggested to modulate signaling by heparin-binding growth factors, and downregulation represents a novel mechanism by which cancer cells can enhance growth factor signaling [53].

Like all complex diseases, CRC is heterogeneous and most likely due to multiple partially penetrant susceptibility alleles as well as non-genetic factors. In order to maximize power to detect linkage, we sought to increase genetic homogeneity by grouping families with similar, potentially genetically driven features, such as age at diagnosis, clinic-based ascertainment, and number of affected family members [5]. A number of other groups have taken a similar predefined subset approach, reporting evidence of CRC linkage in specific regions among family subsets [54]. Here, linked regions on 4q21.1 and 8q13.2 become apparent only in the families with younger mean age at diagnosis and only two affected members, respectively, and the 15q22.31 peak suggested by analysis of all families strengthened considering clinic-based or large families only. Two observations provide particular reassurance of the use of this subset approach: first, the subsets predicted by segregation analysis to be more likely to be genetic (younger age at diagnosis, clinic-based) showed greater evidence for linkage; and second, the peak among smaller families (sibling pairs) was identified using a recessive model.

Other CRC linkage scans have reported evidence of linkage at 3q21-24 and 9q22.2-31.2 in more than one study (Table S3) [18]–[20], [22]–[26], [54]. Evidence of linkage on 3q was first reported in 12 large families with an HLOD score of 3.10 (NPL = 3.40) [20], followed by an independent study of 30 Swedish families at a 65 cM region flanked by markers D3S1558 and D3S3592 on chromosome 3q13.31-27.1 overlapping with the earlier report [24]. Another study that focused on MSS families specifically showed evidence of linkage at this 3q region with an HLOD of 1.49 [26]. Wiesner *et al* [18] identified a linkage peak on chromosome 9q22.2-31.2 region (p = 0.00045) in 53 MSS kindreds in which at least two siblings were diagnosed with colon cancer by age 64 or younger. Subsequently, the linkage peak, flanked by markers D9S283 (80 cM) and D9S938 (104 cM), was narrowed to 7.7 cM by three other studies [21], [22], [25]. In the current study, we detected no linkage in this 9q22 region under either dominant or recessive models. None of the other previously published linkage regions (1p31.1, 4q31.3, 7q31.1, 15q14-22 and 17p13.3 [23], [54]) showed evidence of linkage with HLOD of 2.0 or higher in our study, although some regions harbored HLODs close to 1.0 (Table S3).

A number of factors about this study are unique among CRC genome-wide linkage-scans. First, ours is the largest study, thus had higher power for detection. Second, our population included only families with no evidence of MMR deficiency. Only two smaller studies focused on pMMR families [18], [26]. In this respect, our approach of studying a large number of pMMR families allowed us to identify specific linkage regions for this subgroup of families who are known to differ clinically from dMMR families and do not arise from MMR mutations [55]–[57]. Unlike some prior studies, we included MSI-L families (N = 15) in our analysis, because the relatedness of this phenotype to dMMR disease is unknown; in all regions, results did not differ when analyses were repeated exclusion of these families. Finally, the two most significant regions reported here showed similar NPL scores in these regions.

Several GWAS have reported highly replicated low-penetrance loci [10]–[17], including a meta-analysis of ten independent studies (11,067 cases and 12,517 controls) which replicated eight previously-reported associations [46]. In relation to the four linkage regions reported here, the closest reported GWAS association is on chromosome 12q24 (rs7315438) [46] 3 Mb away for our peak HLOD. It is not surprising that GWAS and linkage analyses may identify different loci due to the complementary strengths of each approach and the evidence, for many cancers, that the familial and non-familial forms of the disease do not often show affected pathways in common. This is largely supported by our analysis of association within the linkage regions reported here. In fact, despite the attractiveness of the two-hit hypothesis, colorectal cancer is an important exception to the pattern among adult cancers, rather than the rule: *APC* is central to a dominant familial syndrome and frequently mutated somatically in the non-familial disease [58]. There is a similar pattern involving the MMR genes: they are mutated in the germline among those with Lynch syndrome, and *MLH1*, at least, is frequently hyper-methylated in the non-familial cancer.

In conclusion, these results suggest novel CRC susceptibility loci on chromosomes 4q21, 8q13, 12q24, and 15q22. Further confirmatory studies are needed, including targeted sequencing and dense mapping of the identified linkage regions. Targeted sequencing of these regions will facilitate identification of novel variants that may be missed with linkage analysis, while fine-mapping studies will narrow the region of interest to be examined. In addition, pooling of linkage data across multiple genome-wide scans should allow for fine-level analysis of discrepant results across family collections. It is clear from this work and the work of others that multiple loci are involved in increasing susceptibility to CRC in families and that family-based studies remain critical to the identification and characterization of these loci.

## Supporting Information

Figure S1 **Ethnicity Estimation using Eigen Analysis.** EIGENSTRAT was used to verify ethnic similarity among related individuals from 544 families based on self-report and to estimate ethnicity for individuals with missing ethnicity. The first two principal components are plotted by (A) Self-reported ethnicity and (B) Genetically-inferred ethnicity which shows a circle surrounding the samples analyzed for linkage.
(DOCX)

Figure S2 **Genome-wide Linkage Scans of White pMMR Family Groups with HLOD<3.0.** Genome-wide linkage scans of three white pMMR family groups with HLODS<3.0. The blue line represents HLODs under the dominant model and the red line represents the HLODs under the recessive model. (A) Family mean age at diagnosis ≥50 years (N = 298). (B) Families with 3 affected members (N = 89). (C) Population-based families (N = 189). (D) Families with unknown ascertainment (N = 79).
(DOCX)

Figure S3 **Regional Association Plot from Population-based Colorectal Cancer Case Control Analysis in 4q21.1.** Plot shows the 1-HLOD interval surrounding rs10518142, the peak linkage SNP among families with younger mean age at diagnosis. The x-axis indicates genomic position. The y-axis indicates −log_10_ association p-values for genotyped SNPs (solid circles) adjusted for age, gender, study site, and four principal components representing ancestry. The most significantly associated SNP is a indicated by a purple diamond. Other than rs10518142 which is indicated by a yellow circle, the colored points indicate the strength of LD with the SNP most associated with CRC risk (purple diamond). Also shown are the SNP build 36 coordinates in kilobases (kb) and a subset of the known genes in the region (below x-axis).
(DOCX)

Table S1 **SNP Exclusions and Number of Analyzed SNPs.**
(DOCX)

Table S2 **Genetic Models Assumed for Parametric Analyses.**
(DOCX)

Table S3 **Comparison to Prior Colorectal Cancer Linkage Studies.**
(DOCX)
