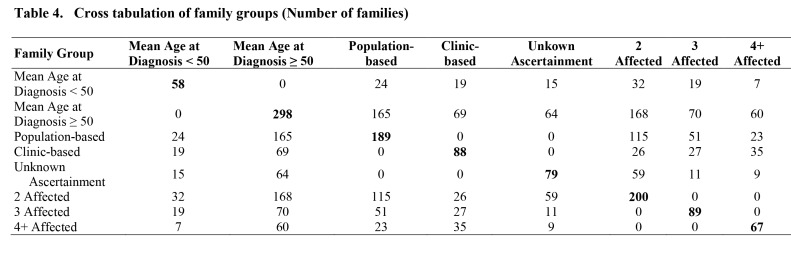# Correction: Colorectal Cancer Linkage on Chromosomes 4q21, 8q13, 12q24, and 15q22

**DOI:** 10.1371/annotation/1ba2f5e3-8aef-4a12-909b-23f95a889325

**Published:** 2012-11-06

**Authors:** Mine S. Cicek, Julie M. Cunningham, Brooke L. Fridley, Daniel J. Serie, William R. Bamlet, Brenda Diergaarde, Robert W. Haile, Loic Le Marchand, Theodore G. Krontiris, H. Banfield Younghusband, Steven Gallinger, Polly A. Newcomb, John L. Hopper, Mark A. Jenkins, Graham Casey, Fredrick Schumacher, Zhu Chen, Melissa S. DeRycke, Allyson S. Templeton, Ingrid Winship, Roger C. Green, Jane S. Green, Finlay A. Macrae, Susan Parry, Graeme P. Young, Joanne P. Young, Daniel Buchanan, Duncan C. Thomas, D. Timothy Bishop, Noralane M. Lindor, Stephen N. Thibodeau, John D. Potter, Ellen L. Goode, for the Colon CFR

There is an error in Table 4. The correct table can be found here: 

**Figure:**