# Supplementary figures and images for: Colorectal Cancer Linkage on Chromosomes 4q21, 8q13, 12q24, and 15q22
Source: PLoS One. 2012 May 31;7(5):e38175. doi: 10.1371/journal.pone.0038175 (PMC3364975; doi:10.1371/journal.pone.0038175)

**Figure S1.** Ethnicity Estimation using Eigen Analysis

**
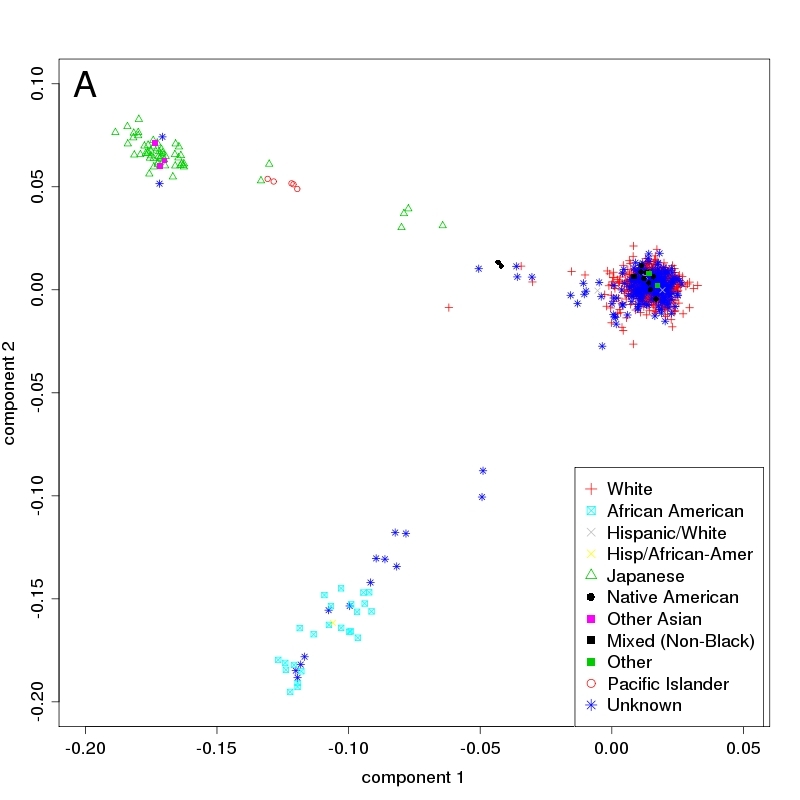

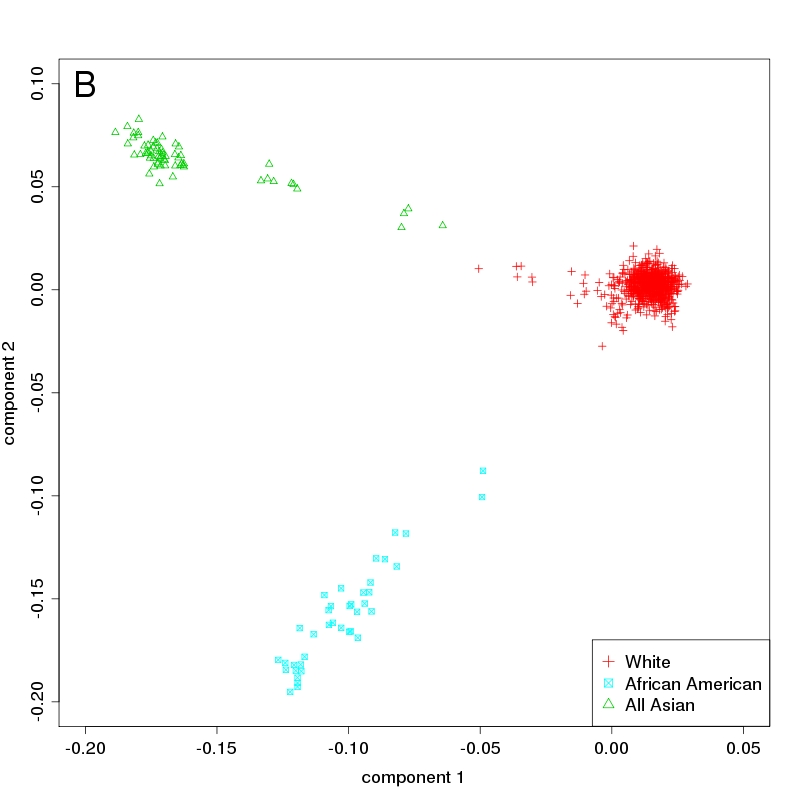
**

Supplement: Figure S1 — Ethnicity Estimation using Eigen Analysis. EIGENSTRAT was used to verify ethnic similarity among related individuals from 544 families based on self-report and to estimate ethnicity for individuals with missing ethnicity. The first two principal components are plotted by (A) Self-reported ethnicity and (B) Genetically-inferred ethnicity which shows a circle surrounding the samples analyzed for linkage. (DOCX) [file pone.0038175.s001.docx]

**Figure S2.** Genome-wide Linkage Scans of White pMMR Family Groups with HLOD < 3.0

**
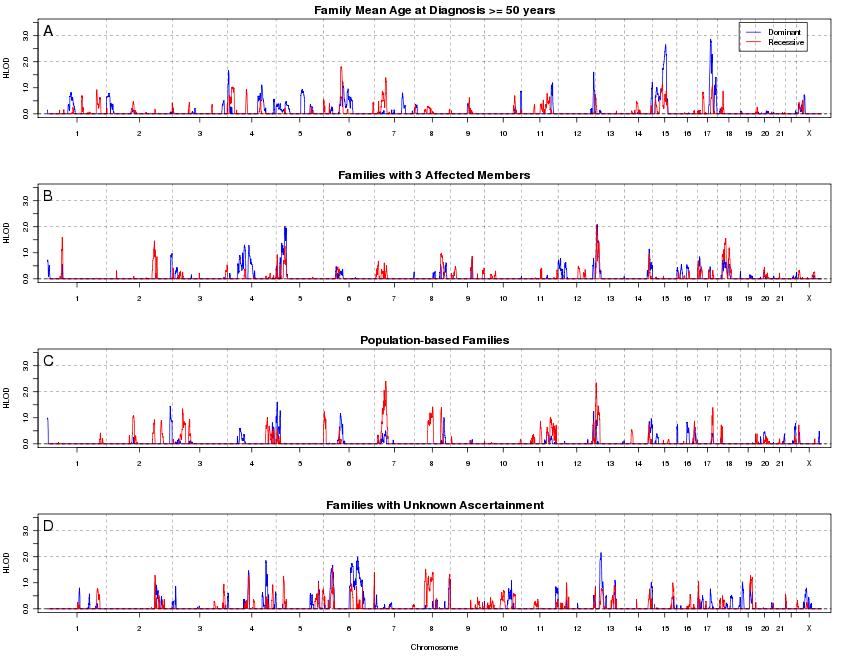
**

Supplement: Figure S2 — Genome-wide Linkage Scans of White pMMR Family Groups with HLOD<3.0. Genome-wide linkage scans of three white pMMR family groups with HLODS<3.0. The blue line represents HLODs under the dominant model and the red line represents the HLODs under the recessive model. (A) Family mean age at diagnosis ≥50 years (N = 298). (B) Families with 3 affected members (N = 89). (C) Population-based families (N = 189). (D) Families with unknown ascertainment (N = 79). (DOCX) [file pone.0038175.s002.docx]
