# Supplementary material for: Colorectal Cancer Linkage on Chromosomes 4q21, 8q13, 12q24, and 15q22
Source: PLoS One. 2012 May 31;7(5):e38175. doi: 10.1371/journal.pone.0038175 (PMC3364975; doi:10.1371/journal.pone.0038175)
Supplement: Figure S3 — Regional Association Plot from Population-based Colorectal Cancer Case Control Analysis in 4q21.1. Plot shows the 1-HLOD interval surrounding rs10518142, the peak linkage SNP among families with younger mean age at diagnosis. The x-axis indicates genomic position. The y-axis indicates −log10 association p-values for genotyped SNPs (solid circles) adjusted for age, gender, study site, and four principal components representing ancestry. The most significantly associated SNP is a indicated by a purple diamond. Other than rs10518142 which is indicated by a yellow circle, the colored points indicate the strength of LD with the SNP most associated with CRC risk (purple diamond). Also shown are the SNP build 36 coordinates in kilobases (kb) and a subset of the known genes in the region (below x-axis). (DOCX) [file pone.0038175.s003.docx]

**Figure S3.** Regional Association Plot from Population-based Colorectal Cancer Case Control Analysis in 4q21.1


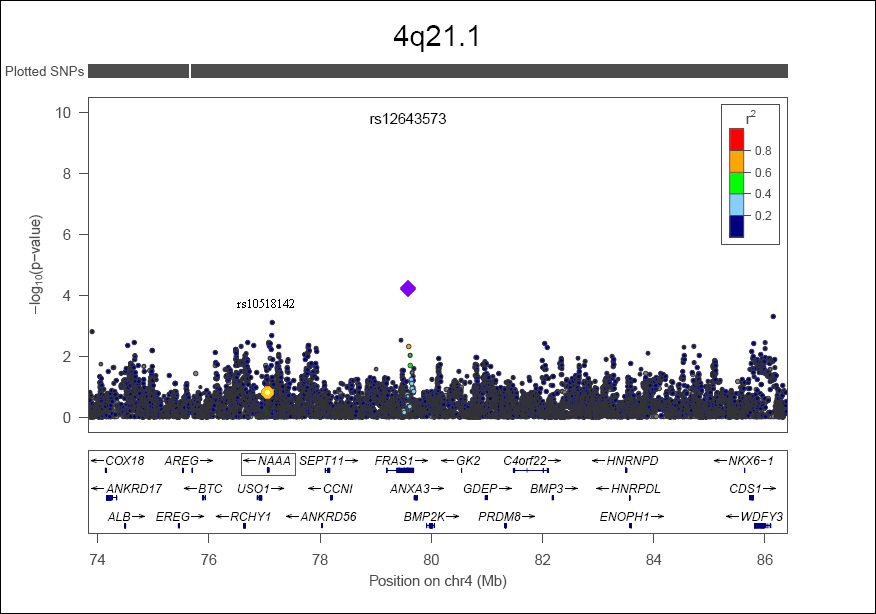

rs10518142

rs12643573
