# Supplementary material for: Colorectal Cancer Linkage on Chromosomes 4q21, 8q13, 12q24, and 15q22
Source: PLoS One. 2012 May 31;7(5):e38175. doi: 10.1371/journal.pone.0038175 (PMC3364975; doi:10.1371/journal.pone.0038175)
Supplement: Table S1 — SNP Exclusions and Number of Analyzed SNPs. (DOCX) [file pone.0038175.s004.docx]

**Table S1.** SNP Exclusions and Number of Analyzed SNPs

|  | **Affymetrix 10K 2.0 Array** | | **Illumina Infinium Linkage 12 Bead Array** | |
| --- | --- | --- | --- | --- |
|  | **Excluded** | **Remaining** | **Excluded** | **Remaining** |
| Total N on array | --- | 10,204 | --- | 6,090 |
| Unknown genetic position^a^ | 114 | 10,090 | 33 | 6,057 |
| Call rate < 95% | 686 | 9,404 | 390 | 5,667 |
| MAF^b^ < 1% | 366 | 9,038 | 11 | 5,656 |
| HWE^c^ p-value < 0.001 | 13 | 9,025 | 4 | 5,652 |
| Duplicate concordance < 95% | 6 | 9,019 | 4 | 5,648 |
| Mendelian errors in 2% of families | 4 | 9,015 | 0 | 5,648 |
| LD^d^ at r^2^ ≥ 0.1 | 3,435 | 5,580 | 1,057 | 4,591 |
| **Final Unique SNPs combined** ^e^**: 10,091** |  |  |  |  |

^a^ NCBI Build 36.3

^b^ Minor allele frequency

^c^ Hardy-Weinberg equilibrium

^d^ Linkage disequilibrium

^e^ An overlapping 80 SNPs in both arrays were included regardless of LD, thus the total of unique SNPs is 80 less than the sum of SNPs remaining in each array.
