# Supplementary material for: Colorectal Cancer Linkage on Chromosomes 4q21, 8q13, 12q24, and 15q22
Source: PLoS One. 2012 May 31;7(5):e38175. doi: 10.1371/journal.pone.0038175 (PMC3364975; doi:10.1371/journal.pone.0038175)
Supplement: Table S2 — Genetic Models Assumed for Parametric Analyses. (DOCX) [file pone.0038175.s005.docx]

**Table S2.** Genetic Models Assumed for Parametric Analyses

|  |  | **Recessive Model (q=0.049)** | | | |  | **Dominant Model (q=0.0046)** | | | |
| --- | --- | --- | --- | --- | --- | --- | --- | --- | --- | --- |
| **Liability Class ^b^** | **Disease State, Age** | **Penetrances ^a^** | | | **Penetrance** |  | **Penetrances ^a^** | | | **Penetrance** |
|  |  | **dd** | **dD** | **DD** | **Ratio ^c^** |  | **dd** | **dD** | **DD** | **Ratio ^c^** |
| 1 | Affected, 20 – 29 | 0.00009 | 0.00009 | 0.013 | 144.4 |  | 0.00009 | 0.003 | 0.003 | 33.3 |
| 2 | Affected, 30 – 39 | 0.00031 | 0.00031 | 0.042 | 135.5 |  | 0.00031 | 0.007 | 0.007 | 22.6 |
| 3 | Affected, 40 – 49 | 0.0016 | 0.0016 | 0.181 | 113.1 |  | 0.0016 | 0.032 | 0.032 | 20.0 |
| 4 | Affected, 50 – 59 | 0.007 | 0.007 | 0.368 | 52.6 |  | 0.007 | 0.096 | 0.096 | 13.7 |
| 5 | Affected, 60 – 69 | 0.018 | 0.018 | 0.296 | 16.4 |  | 0.018 | 0.171 | 0.171 | 9.5 |
| 6 | Affected, 70 – 79 | 0.034 | 0.034 | 0.05 | 1.5 |  | 0.034 | 0.091 | 0.091 | 2.7 |
| 7 | Affected, 80+ | 0.051 | 0.051 | 0.05 | 1.0 |  | 0.051 | 0.1 | 0.1 | 2.0 |
| 8 | Unaffected, 20 – 29 | 0.00009 | 0.00009 | 0.013 | 144.4 |  | 0.00009 | 0.003 | 0.003 | 33.3 |
| 9 | Unaffected, 30 – 39 | 0.0004 | 0.0004 | 0.055 | 137.5 |  | 0.0004 | 0.01 | 0.01 | 25.0 |
| 10 | Unaffected, 40 – 49 | 0.002 | 0.002 | 0.236 | 118.0 |  | 0.002 | 0.042 | 0.042 | 21.0 |
| 11 | Unaffected, 50 – 59 | 0.009 | 0.009 | 0.604 | 67.1 |  | 0.009 | 0.138 | 0.138 | 15.3 |
| 12 | Unaffected, 60 – 69 | 0.027 | 0.027 | 0.9 | 33.3 |  | 0.027 | 0.309 | 0.309 | 11.4 |
| 13 | Unaffected, 70 – 79 | 0.061 | 0.061 | 0.95 | 15.6 |  | 0.061 | 0.4 | 0.4 | 6.6 |
| 14 | Unaffected, 80+ | 0.112 | 0.112 | 1 | 8.9 |  | 0.112 | 0.5 | 0.5 | 4.5 |
| 15 | Affected/Unaffected, <20 | 0 | 0 | 0 | 1.0 |  | 0 | 0 | 0 | 1.0 |

^a^ Allele D is the CRC disease allele with frequency = q. Penetrances represent the probability that an individual with the given genotype will be affected.

^b^ For liability classes 1-7, this is the probability of becoming affected during the age group shown, and for liability classes 8-14, this is the probability of becoming affected before or during the age group shown.

^c^ Penetrance ratio = penetrance of DD genotype ÷ penetrance of dd genotype.
