# Supplementary material for: Colorectal Cancer Linkage on Chromosomes 4q21, 8q13, 12q24, and 15q22
Source: PLoS One. 2012 May 31;7(5):e38175. doi: 10.1371/journal.pone.0038175 (PMC3364975; doi:10.1371/journal.pone.0038175)
Supplement: Table S3 — Comparison to Prior Colorectal Cancer Linkage Studies. (DOCX) [file pone.0038175.s006.docx]

**Table S3.** Comparison to Prior Colorectal Cancer Linkage Studies

| **Region** | **Marker (cM^a^)** | **Results** | **Population^b^ / N Families** | **Ref.** | **Present Result in 356 Families** |
| --- | --- | --- | --- | --- | --- |
| 3q21-q24 | rs718612 - rs966226  (239.88 cM - 275.34 cM) | HLOD=3.10, α=0.62  NPL=3.40 | CoRGI UK / 69 | [^20^](#_ENREF_20) | Dominant HLOD = 0.51 (350.43 cM)  Recessive HLOD = 0.05 (280.51 cM) |
| 3q21.1-q26.2 | D3S1558 - D3S3592  (233.4 cM - 362.7 cM) | HLOD=1.9, α=0.45  NPL=2.1 | FCC Sweden / 30 | [^24^](#_ENREF_24) | Dominant HLOD = 0.51 (350.43 cM)  Recessive HLOD = 0.05 (280.51 cM) |
| 3q21.3 | NA**^c^** | HLOD=1.49, α=0.57  NPL=3.65 | Netherlands / 7 | [^26^](#_ENREF_26) | Dominant HLOD = 0.51 (350.43 cM)  Recessive HLOD = 0.05 (280.51 cM) |
| 7q21.12 | D7S2195 - D7S3058  (273.4 cM - 312.8 cM) | p=0.0011 | CNSS / 194 | [^55^](#_ENREF_55) | Dominant HLOD = -0.16 (310.43 cM)  Recessive HLOD = -1.03 (293.45 cM) |
| 7q31.31 | D7S643 (216 cM) | NPL=3.08 | CGN / 70 | [^23^](#_ENREF_23) | Dominant HLOD = 0.26 (216.0 cM)  Recessive HLOD = -1.18 (216.0 cM) |
| 9q22.2-31.2 | D9S283 - D9S938  (156.92 cM - 187.8 cM) | p=0.00045 | CNSS / 53 | [^18^](#_ENREF_18) | Dominant HLOD = -0.35 (170.9 cM)  Recessive HLOD = 1.22 (157.15 cM) |
| 9q22.33 | D9S971 – D9S272/D9S173  (171.0 cM - 176.1 cM) | HLOD=1.23, α=0.20  NPL=1.21 | CoRGI UK/ 57 | [^21^](#_ENREF_21) | Dominant HLOD = -0.73 (176.13 cM)  Recessive HLOD = -0.41 (176.13 cM) |
| 9q22.32-31.1 | D9S280 – D9S277  (170.2 cM - 185.7 cM) | HLOD=2.4 | FCC Sweden / 1 | [^22^](#_ENREF_22) | Dominant HLOD = -0.35 (170.9 cM)  Recessive HLOD = 0.75 (170.24 cM) |
| 9q22 | D9S1815 – D9S1857  (165.1 cM - 176.1 cM) | p=0.0001 | CNSS+CCFR / 56+54 | [^25^](#_ENREF_25) | Dominant HLOD = -0.35 (170.9 cM)  Recessive HLOD = 0.77 (166.28 cM) |
| 11q23 | D11S1314 – D11S908 (115.2 cM - 183.2 cM) | HLOD=1.96-2.10, α=0.25-0.35  NPL=1.28-2.16 | Sweden / 18 | [^19^](#_ENREF_19) | Dominant HLOD = -0.70 (147.94 cM)  Recessive HLOD = 0.24 (150.78 cM) |

^a^ All the genetic distances are reported in Haldane cM.

^b^ FCC – Family Cancer Clinic at the Karolinska Hospital, Stockholm, Sweden; CNSS – Colon Neoplasia Sibling Study; CGN – Cancer Genetics Network; CoRGI – ColoRectal Tumor Gene Identification Study Consortium, UK

^c^ Markers are not specified for this region in the corresponding report.
